# Angle-insensitive co-polarized metamaterial absorber based on equivalent circuit analysis for dual band WiFi applications

**DOI:** 10.1038/s41598-021-93322-5

**Published:** 2021-07-02

**Authors:** Saif Hannan, Mohammad Tariqul Islam, Mohammad Rashed Iqbal Faruque, Muhammad E. H. Chowdhury, Farayi Musharavati

**Affiliations:** 1 Department of Electrical, Electronic and Systems Engineering, Faculty of Engineering and Built Environment, Universiti Kebangsaan Malaysia, 43600 Bangi, Selangor Malaysia; 2 Space Science Center (ANGKASA), Universiti Kebangsaan Malaysia, 43600 Bangi, Selangor Malaysia; 3 Department of Electrical Engineering, Qatar University, 2713 Doha, Qatar; 4 Mechanical and Industrial Engineering Department, Qatar University, 2713 Doha, Qatar

**Keywords:** Engineering, Materials science

## Abstract

A novel and systematic procedure to design a co-polarized electromagnetic metamaterial (MM) absorber with desired outputs and resonance frequencies for dual-band WiFi signal absorption is presented. The desired resonance frequencies with expected S parameters' values were first designed as an equivalent circuit with extensive analysis and then implemented into frequency-selective MM absorber by numerical simulation with precise LRC elements, satisfying least unit cell area (0.08λ), substrate thickness (0.01λ) and maximum effective medium ratio (12.49). The absorber was simulated for the maximum angle of incidence for both the normal and oblique incidences at co-polarization. The absorptions at the desired resonance frequencies were found at a satisfactory level by both simulation and practical measurement along with a single negative value to ensure metamaterial characteristics. The proposed equivalent circuit analysis approach can help researchers design and engineering co-polarization insensitive MM absorbers using conventional split-ring resonators, with perfection in output and desired resonance frequencies without the necessity of lumped elements or multilayer substrates. The proposed metamaterial can be utilized for SAR reduction, crowdsensing, and other WiFi-related practical applications.

## Introduction

Metamaterial (MM) absorbers are an engrossing subject among engineers and scientists for a wide variety of applications in electromagnetic, acoustic, optical, and mechanical engineering. Research works are being done for designing perfect and efficient MM absorbers for EM wave applications since the last decade. Hundreds of designs have been done to date for microwave MM absorbers, a few of which are perfect MM absorbers^1^ considering insensitivity to co- & cross-polarization and rotational symmetry^2–4^. Moreover, almost all the absorbers are not perfect due to cross-polarization insensitivity due to their structural design^5,6^. The usual split-ring resonator (SRR), complementary split-ring resonators (CSRR), or coupled—non-coupled SRRs are the most common type of resonators designed for these purposes^7–9^, which are popular due to simplicity in structural design and easiness of explanation. These SRRs or CSRRs or coupled SRRs are mostly co-polar MM absorbers^10^, despite some are polarization converters^11^.

The so-called co-polarization insensitive MM absorbers are being designed by random patch designs for single negative (SNG) or double negative (DNG) values of the permittivity and the permeability with negative or near zero value of the refractive index^12–14^. The resonance frequencies found from simulation and measurements afterward could never be adequately explained, let alone explain the size of the unit cell, size and shape of the patch, and the width of the dielectric substrate for the achieved resonance frequencies or absorptions. In other words, none of these co-polar absorbers were designed targeting a particular or several desired frequencies that could be utilized for practical or purpose-wise applications for real-world needs. It seems apparent that there must be some technique and proper explanation to design a MM absorber at desired frequencies with desired values of S parameters, which can be explained logically.

To date, no such works are found that can logically explain the patch structure for having its resonance frequencies or S parameters (which can explain SNG or DNG properties; as a result, the MM characteristics), which eventually makes us blind towards understanding the MM characteristic-reason of the patch structure. Hence, there must be a logical and systematic process to design co-polar MM absorbers with target resonances and desired S parameters values so that the MM characteristic can be correlated with the patch design. Equivalent circuit modeling of frequency selective surfaces (FSS) of metamaterials is such an idea that can explain the metamaterial behaviour of the structure^15,16^. Few articles have been published describing equivalent circuit analysis for better interpretation of the designed absorber^16–26^, but no papers are found for WiFi signal absorber with necessary equivalent circuit analysis.

This paper proposes a systematic method to design a co-polarization insensitive metamaterial absorber with desired S parameters and resonance frequencies for WiFi signal (2.4 GHz and 5 GHz) absorption with extensive equivalent circuit analysis. The unit cell of the proposed absorber has been developed following an equivalent circuit. Conventional square-shaped split-ring resonators (SRR) have been used for perfect designing of the absorber with estimated unit cell size and patch dimensions to avoid unwanted complexities such as capacitive coupling due to intricate patch design and phase mismatching. For simplicity and better understanding, this article has been organized with sections describing transmission line model, design of the equivalent circuit, estimation of required resistances for desired values of S parameters from the circuit, management of the FSS for dual-band absorption, performance analysis of the designed unit cell, measurement of the fabricated absorber and discussion, respectively. Finally, a conclusion and comparing the proposed absorber's performance indicators with other WiFi absorbers have established the proposed design as the best WiFi absorber that comprehends with appropriate equivalent circuit analysis.

### Transmission line general model for the co-polarized absorber

**Figure 1:**
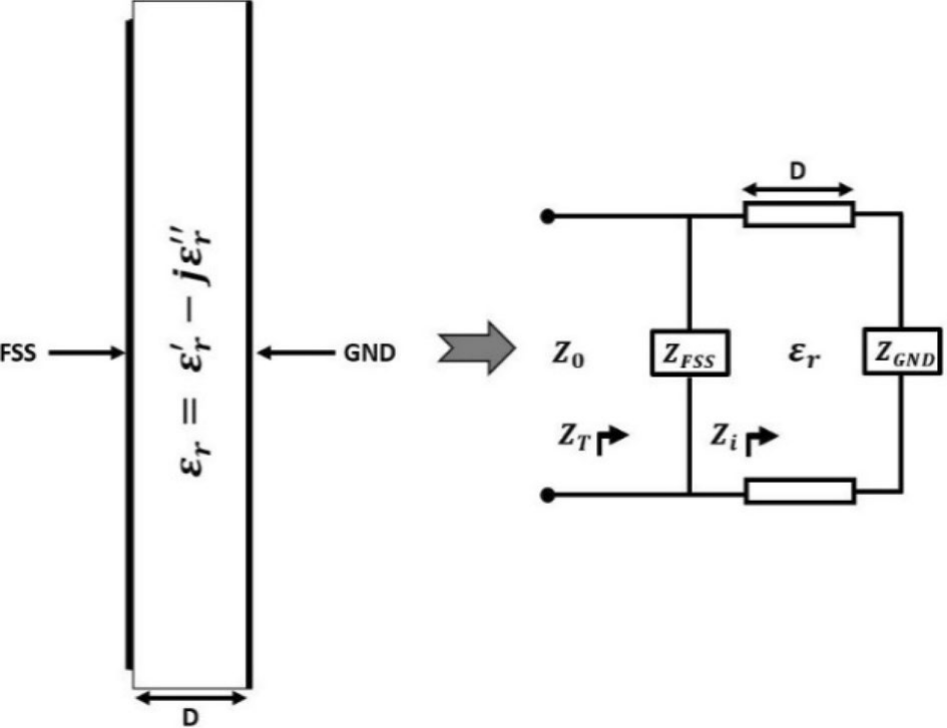
Right side view of the proposed metamaterial absorber and equivalent transmission line model.

The transmission line model and the right-sided view of the metamaterial structure with three layers (patch, substrate, and ground) are shown in Fig. 1 below. The dielectric substrate (of thickness D) of the structure is represented in the transmission line by “D.” The FSS (patch) impedance, $Z_{{FSS}}$ is connected to one end of the transmission line, and the other end is associated with the partial metallic ground of impedance $Z_{{GND}}$. The metal ground will have a cut area at the center to act as a stub for impedance matching, which should be equal to the characteristic impedance $Z_{0} \left( { = \sqrt {\frac{L}{C}} } \right)$ of the metamaterial.

The input impedance $Z_{i}$ of the metamaterial for the matched transmission line at the ground plane can be expressed as:

1 $$ Z_{i}  = j\frac{{Z_{0} }}{{\sqrt {\varepsilon _{r} } }}\tan \left( {k_{0} \sqrt {\varepsilon _{r} } ~D} \right) $$

where $~\varepsilon _{r} \left( { = ~\varepsilon _{r}^{'}  - j\varepsilon _{r}^{{''}} } \right)$ is the permittivity of the dielectric substrate and $k_{0}$ is the wave propagation constant.

The total impedance $Z_{T}$ of the metamaterial^27^ must be compatible with free space impedance $Z_{0}$ in order to minimize reflection and transmission of applied EM wave from the metamaterial.

2 $$ Z_{T}  = ~Z_{{FSS}} ~||~\left( {Z_{D}  + Z_{{GND}} } \right) $$

So that, $S_{{11}}  = ~\frac{{Z_{T}  - Z_{0} }}{{Z_{T}  + Z_{0} }}$ and $S_{{21}}  = ~\frac{{2~Z_{T} }}{{Z_{T}  + Z_{0} }}$. The structure's reflection and transmission coefficients should be zero for perfect absorption. Therefore $Z_{T}$ must obey the conditions as (i) *Re* ($Z_{T}$) $\approx Z_{0}$ and (ii) *Im* ($Z_{T}$) with the smoothed transition from zero line.

### Proposed dual-band absorber with equivalent circuit model

The first step to designing an absorber unit cell with desired resonances and S parameters is to get L, C, and R of the individual microstrip SRRs on the patch with precision values for each resonance frequency. These precise values can be achieved from an equivalent circuit model. The equivalent circuit can be designed by commercially available software like ADS^28^.

#### Determining L and C for resonance Frequencies

**Figure 2:**
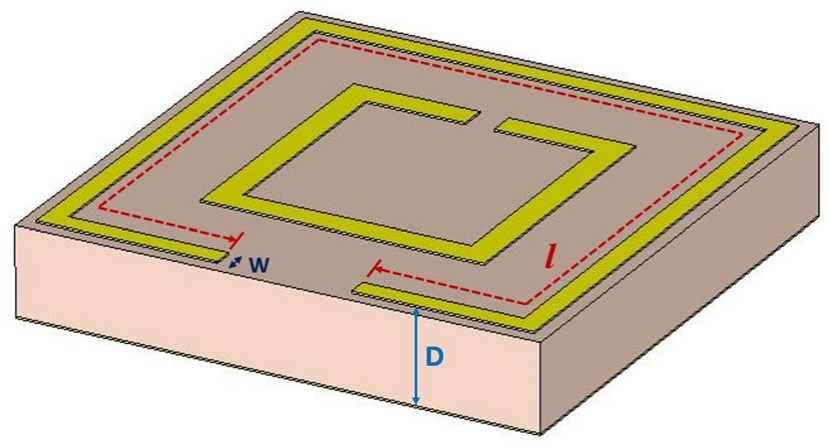
Microstrip resonator in the MM unit cell patch.

Each resonator of the MM absorber unit cell patch has an inductive value, which can be determined^29^ by the following Eq. (3)^30^ as per Fig. 2.

3 $$ L_{{ms}}  = 0.00508L~\left[ {\ln \left( {\frac{{2l}}{{W + D}}} \right) + 0.5 + 0.2235~\left( {\frac{{W + D}}{l}} \right)} \right]$$

where $L_{{ms}}$ is the inductance per unit length of the microstrip $\left( {\mu H} \right)$, *l* is the length of the strip (inches), W is the width of the strip (inches), and D is the distance between the stripline and the ground plane.

The length and width of the microstrip SRR can be obtained from Eq. (3). The corresponding value of capacitance for the desired resonance frequency^31^ can be obtained from Eq. (4).

4 $$ C = ~\frac{1}{{4\pi ^{2} f^{2} L_{{ms}} }}$$

where $f$ is the desired resonance frequency. For each desired frequency, L and C's required values can be obtained from Eqs. (3) & (4) to use in the circuit simulator.

**Figure 3:**
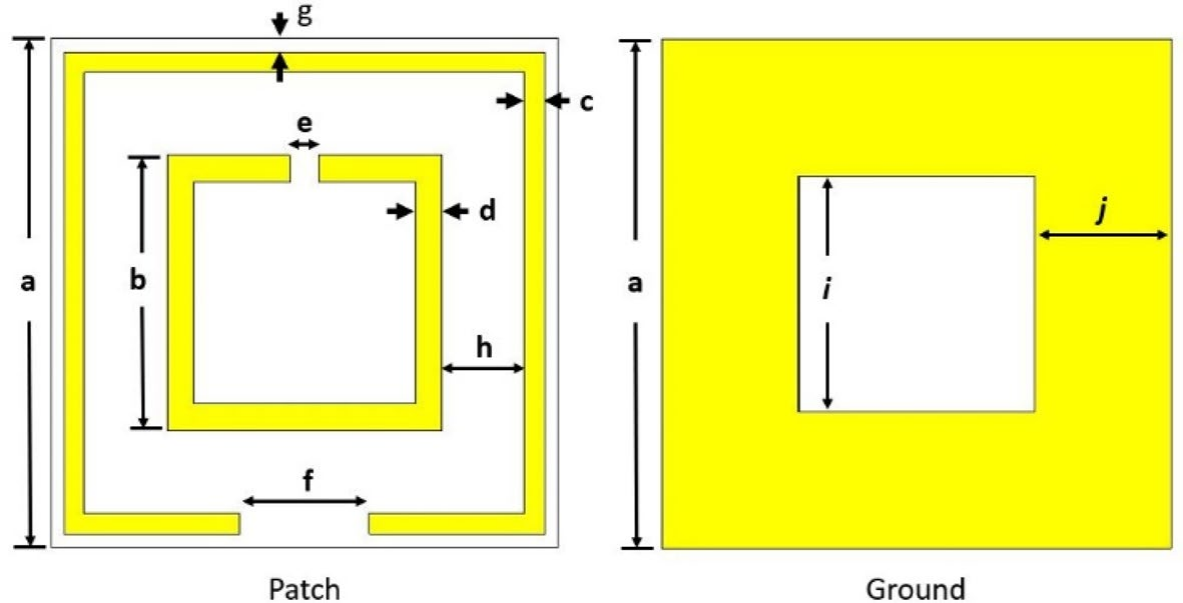
The patch and ground dimensions: a = 9.5 mm, b = 5.14 mm, c = 0.38 mm, d = 0.5 mm, e = 0.53 mm, f = 2.42 mm, g = 0.25 mm, h = 1.55 mm, i = 5.1 mm, j = 2.55 mm.

The structure of the proposed dual-band WiFi signal absorber is depicted in Fig. 3.

The length *l* and width *W* of the both resonator split-rings were decided as per Eq. (3) for 2.4 GHz and 5 GHz, respectively. After that, the desired values of C’s (C1 & C2) for the resonance frequencies were determined as per Eq. (4), which were implemented in the patch using Eq. (5) where the capacitive gaps were calculated as split-gaps (*e* & *f* in Fig. 3). The length, width, and split -gap of the first or the circumferential resonator (for 2.4 GHz) were set first and then the dimensions of the second or the inner resonator (for 5 GHz) and the non-copper ground cut were set considering non-metal surface gaps bounded due to the both the split-rings and the ground cut individually to maintain isolation between the resonance frequencies. Isolation refers to less or no absorption in comparison with maximum absorptions at resonance frequencies. To maintain the isolation, capacitive elements (C4, C5 & C6) were added in among the transmission lines and the ground transmission line.

On the ADS simulator, L and C's values must be put for each resonance frequency as parallel transmission lines connected by capacitive element between them due to the capacitive gaps in between the resonators, as can be seen from Fig. 6c,d. No inductive elements can be added in place of capacitive gaps, since a very large amount of inductivity may require which will deactivate the major inductors (L1 & L2) due to series connection and thus deviate the resonance frequencies. The capacitive gaps due to nonmetal area bounded by the all split-rings and the central ground cut can be estimated from the following Eq. (5) as

5 $$ C = ~\varepsilon _{0} \varepsilon _{r} ~\frac{A}{d}~~ $$

where $\varepsilon _{0}$ is the permittivity of free space (or air), $\varepsilon _{r}$ is the relative permittivity of the substrate, A is the area of the split conducting strip and d (h for outer resonator, (b-2d) for the inner resonator and *i* for the ground cut; in Fig. 3) is the distance for the splits/gaps or the ground cut.

**Figure 4:**
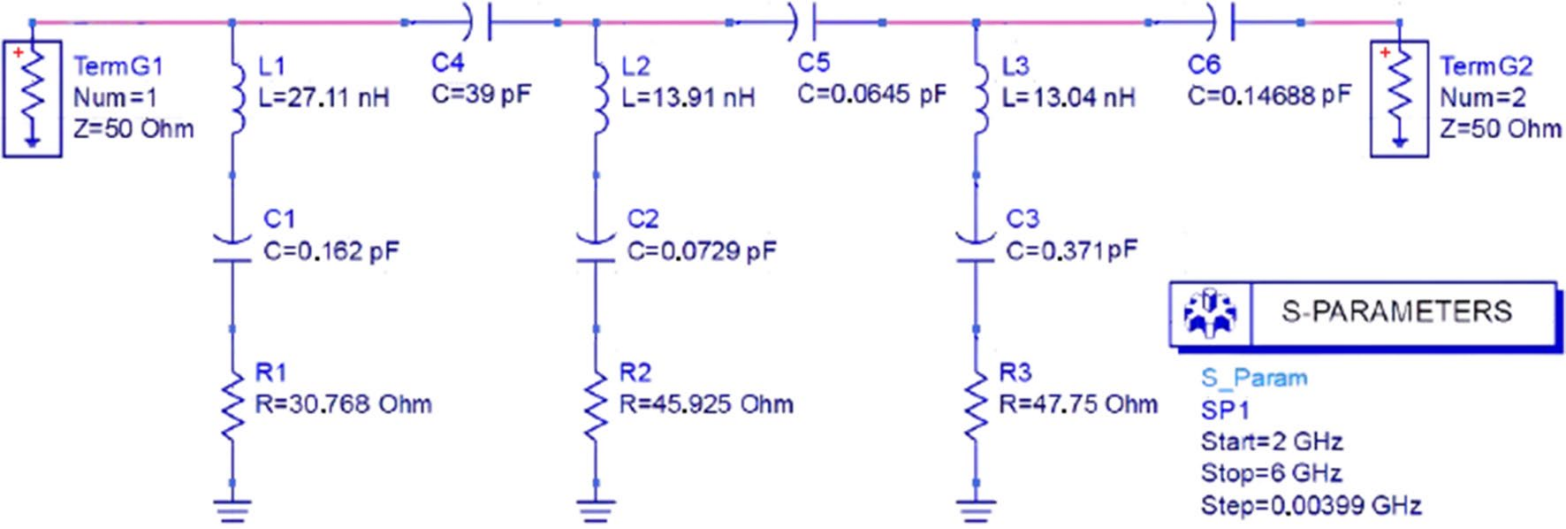
Equivalent circuit of the absorber unit cell.

The unit cell in Fig. 2 was designed to absorb WiFi signals at 2.4 GHz and 5 GHz. Two conventional square SRRs have been used for this purpose. The outer SRR acts for low resonance frequency (2.4 GHz), and the inner SRR works for higher resonance frequency (5 GHz). Each resonator has inductance and self-impedance (R). The values of L and C were set on the ADS circuit, as shown in Fig. 4. The calculated values of inductance and capacitance for 2.4 GHz and 5 GHz resonances are L1 = 27.11 nH, C1 = 0.162 pF and L2 = 13.91 nH, C2 = 0.0729 pF respectively. For the ground, the corresponding values are found as L3 = 13.04 nH, C3 = 0.371 pF.

The first transmission line, L1-C1-R1, stands for lower resonance frequency (2.4 GHz) in Fig. 4. The second line, L2-C2-R2, works for higher resonance frequency (5 GHz) along with the third line L3-C3-R3, which comes out of the ground plane. These parallel segmented transmission lines cannot ensure distinct resonances at 2.4 GHz and 5 GHz since there should be some mutual coupling among them. To reduce these couplings, no inductive element can be added in between the neighboring lines as it will deviate the resonance frequencies due to the added values. Thus, capacitive elements were introduced in series in between parallel neighboring transmission lines, and it can be justified by the capacitive gaps due to non-metal surfaces bounded by the split-rings as shown in Fig. 3. C4 was introduced in between the first and the second line; similarly, C5 in between the second and the third line (Fig. 4). As the metamaterial absorbers are considered periodic structures, the electromagnetic coupling may occur due to inductive and capacitive elements in the circuit, which can be eliminated by introducing series capacitances (like C6) in the circuit, as shown in Fig. 4. Inductive elements in place of C6 cannot be added in the circuit, since the inductive elements will be in series with the ground transmission line (L3-C3-R3) which will deviate the resonance frequencies due to added inductance and thus will fail the intension of the design.

The value of C4 (= 39 pF) was chosen higher than C5 (= 0.0645 pF) and C6 (0.14668 pF), since C4 is considered for due to the non-metal area bounded by the circumferential or outer or first split-ring. The outer or first split-ring influences not only the inner area bounded by it but also the neighboring area around it up to the next unit cell. The values of C4, C5 and C6 were fine-tuned on the ADS circuit simulator until achieving optimum isolation in the areas among resonance frequencies.

#### Estimation of R of each SRR by the equivalent circuit

**Figure 5:**
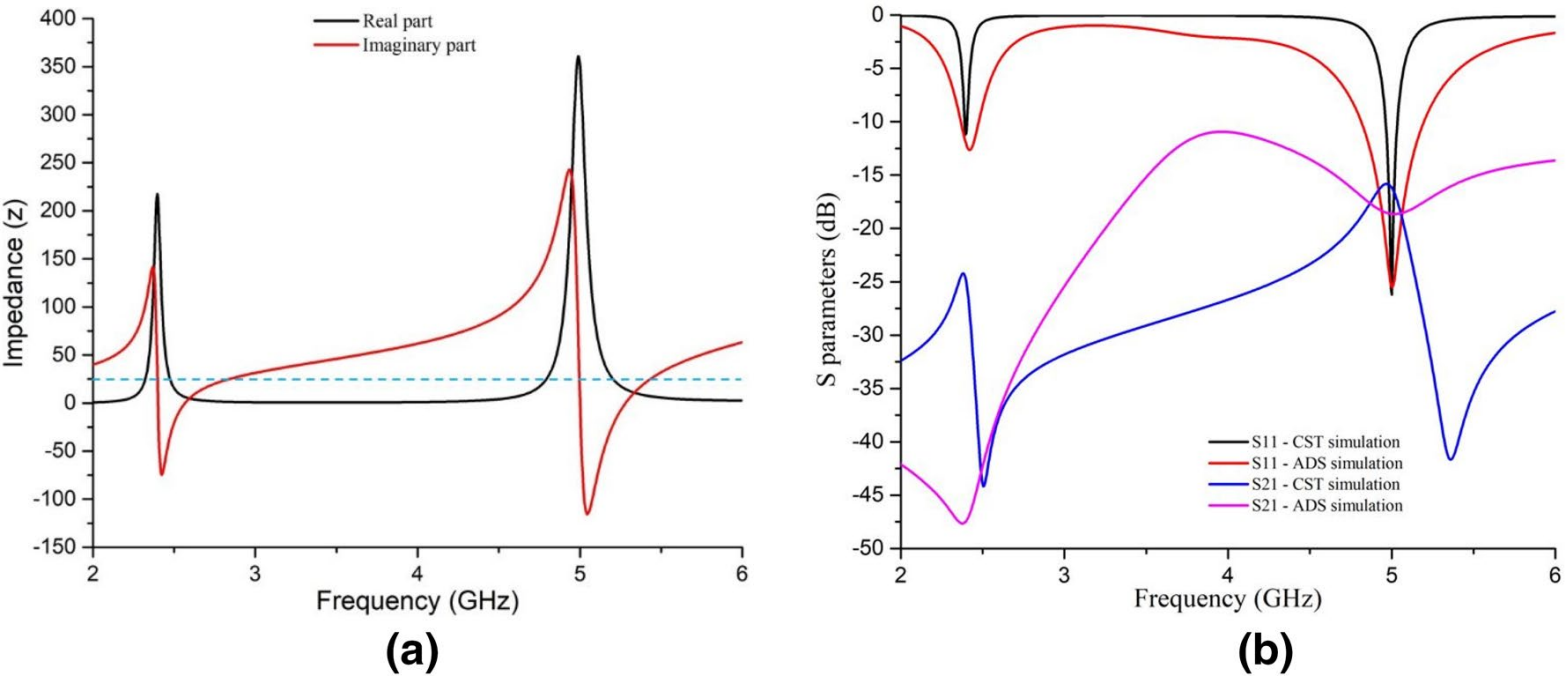
(**a**) impedance of the unit cell at operating frequencies and (**b**) S parameters with less than − 10 dB values achieved by equivalent circuit and CST simulation.

**Figure 6:**
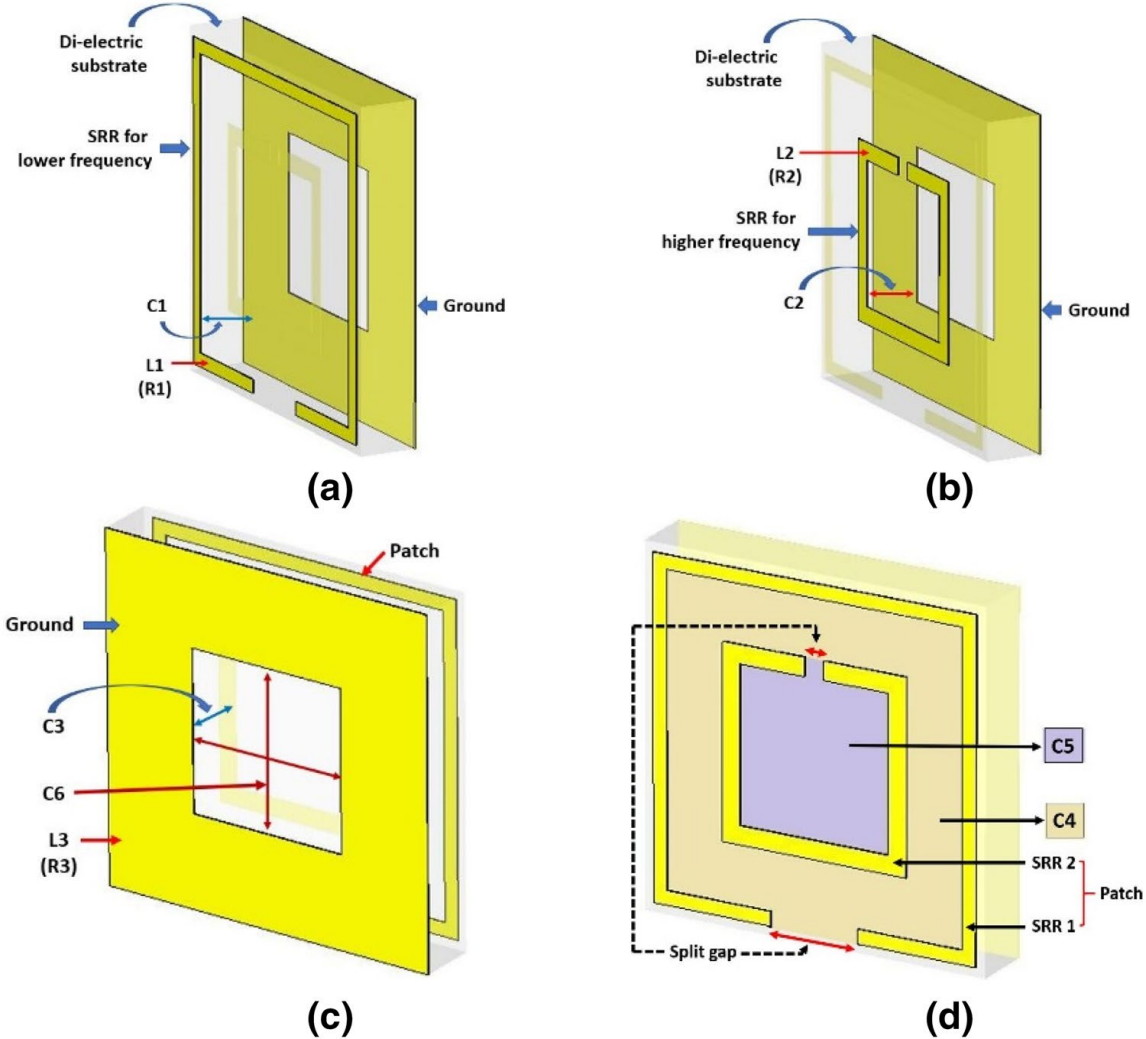
A detailed impression of the L, C, and R in (**a**) the split-ring resonator (SRR) -1 for lower frequency, (**b**) the SRR -2 for the higher frequency, (**c**) the ground; and (**d**) the parallel capacitance due to the bounded surface area by the SRRs.

The mutual coupling between the second and third line tends the value of S11 towards zero in the mid-area between 2.4 GHz and 5 GHz in Fig. 5. The parallel capacitors C4, C5, and C6, are due to plane surfaces bounded by the resonator 1,2, and the ground (as shown in Fig. 6), which sharpens the resonance peaks in Fig. 5. The input impedance of both the waveguide ports was taken 50 ohms by default. To get desired values of S11 and S21 parameters. (in dB), a series of resistance has been added with each LC transmission line. By tuning these resistances, desired dB values of S parameters were achieved, depicted in Fig. 5.

As the unit cell have losses (in terms of R) due to ohmic loss ($$R_{o} )$$ of the area bounded by SRRs and dielectric loss ($$R_{d} )$$ of the substrate, $$R_{o}$$ should be estimated to calculate the desired input impedance of the unit cell structure.

6 $$ R_{o}  = ~\frac{{{\text{Cell~area}}}}{{{\text{metallic~area~of~the~SRR}}}}~\left( {\frac{1}{{\delta \sigma }}} \right) $$

Here, $$\delta$$ is the skin depth, and $$\sigma$$ is the conductivity of the metallic SRR, and $${\raise0.7ex\hbox{$1$} \!\mathord{\left/ {\vphantom {1 {\delta \sigma }}}\right.\kern-\nulldelimiterspace} \!\lower0.7ex\hbox{${\delta \sigma }$}}$$ is the surface resistance due to the non-metal area bounded by the resonator. As surface resistance influences the absorbing properties of the unit cell, it should be correlated with the total resistance (for each resonance frequency) as:

7 $$ R = ~R_{0} ~\left( {\frac{P}{S}} \right)^{2} $$

P is the periodicity of the unit cell, and S is the resonator's exterior length. The dielectric loss ($R_{d}$) can be easily obtained from simulation data that happens due to the unloaded capacitance of the SRR. $R_{d}$ was considered as a variable resistor to avoid the complexity of calculation, which can be tuned on the simulator to get desired S parameters. Since the quasi-static analysis is employed in the equivalent circuit designing, thus effective dielectric constant was considered instead of $R_{d}$. The impedance due to SRR-1 and SRR-2 are $Z_{{SRR1}}$ and $Z_{{SRR2}}$ which can be obtained as:

$$ \begin{aligned}    & Z_{{SRR1}}  = R_{{SRR1}}  + jX \\     & {\text{And}}\;Z_{{SRR2}}  = R_{{SRR2}}  + jX \\  \end{aligned} $$

where $$X\left( { = ~j\omega L + {\raise0.7ex\hbox{$1$} \!\mathord{\left/ {\vphantom {1 {j\omega C}}}\right.\kern-\nulldelimiterspace} \!\lower0.7ex\hbox{${j\omega C}$}}} \right)$$ is coupling reactance appeared on the circuit due to coupling of inductance and capacitance among the SRRs during the absorber's resonating condition. The estimation of R considering reflection^25^ and transmission coefficient can be understood from the following equations as:

8 $$ \left. R \right|_{{min}} ~ < ~Z_{0} ~\frac{{1 - \left| {S_{{11}} } \right|^{2}  - \left| {S_{{21}} } \right|^{2} }}{{1 + \left| {S_{{11}} } \right|^{2}  + \left| {S_{{21}} } \right|^{2} }} $$

9 $$ \left. R \right|_{{max}} ~ < ~Z_{0} ~\frac{{1 + \left| {S_{{11}} } \right|^{2}  + \left| {S_{{21}} } \right|^{2} }}{{1 - \left| {S_{{11}} } \right|^{2}  - \left| {S_{{21}} } \right|^{2} }} $$

Considering Eqs. (7), (8), and (9), the values of R for 2.4 GHz and 5 GHz resonance frequencies were found 30.768 Ohm and 45.925 Ohm for the surfaces bounded by the outer and inner SRR, respectively. The surface framed by the ground metal at its center was 47.74 Ohm to match the total impedance shown in Eq. (2) and Fig. 5a. The total impedance ($Z_{T}$) of the unit cell from simulation (Fig. 5a) shows values near to free space impedance ($Z_{0}$) at resonance frequencies, which satisfies Eq. (2). By tuning the R of each LRC segment, S parameters' desired values were obtained at the resonance frequencies, as depicted in Fig. 5b.

It can be seen from Fig. 5b that, S_11_ parameters found from both CST and ADS simulation coincides at the resonance frequencies and slightly differs at all other frequencies. This difference is negligible since the values of S_11_ parameter found from both the simulations lies in the region greater than − 10 dB and tends to zero, which is definitely not a concern for absorption at all other frequencies except at the resonance frequencies. It is also observable from Fig. 5b that, S_21_ parameters found from both simulations lies less than − 10 dB, but there is some deviation of this parameter from the CST simulation with that of the ADS simulation. This happened because of the mismatch in considering the L-C-R parameters for the ground in the equivalent circuit and the ground designed in the CST simulation. It is difficult to assume L-C-R parameters of the ground precisely for the equivalent circuit under the influence of many factors like dielectric constant of the substrate, mutual capacitive coupling of the metal ground with patch through the substrate and the capacitive non-metal cut at its center. This problem is considered negligible, as S_21_ parameters found from both simulations lies in the region less than − 15 dB, specifically at the resonance frequencies. As the less the values are in dB, the less are the equivalent linear values; and thus, S_21_ parameters in this case has negligible influence on absorption performance.

#### Managing patch and ground dimensions as per equivalent circuit

The unit cell was modified with the dimensions of SRRs, and the ground cut along with the spacing between the SRRs on the patch area. The length and width of the SRRs were calculated following Eq. (3).

Each SRR has inductance along with characteristic impedance^32^. The associated capacitance due to the dielectric substrate in between the SRRs and the ground are C1 and C2 (shown in Fig. 6a,b). The ground itself has capacitance (C3) with the patch (SRRs) (shown in Fig. 6c), as two waveguide ports were applied on both the patch and the ground. Moreover, the SRRs have surface capacitance (C4 & C5) due to their bounding surface area, as shown in Fig. 6d. To set the resonance frequencies at 2.4 GHz and 5 GHz, the ground was cut at its center and tuned with the appropriate area, which introduced a surface capacitance (C6) bounded by the cut area (shown in Fig. 6c).

The gaps (split-gaps) of the split-ring resonators (SRRs) were adjusted until resonance peaks were achieved at desired frequencies, as can be seen from Fig. 6d. This is the essential step to do modification on the SRRs to get desired resonances. Moreover, the cut at the center of the ground plane is the last step to adjust the capacitance C6 (in Fig. 6c) so that the higher resonance frequency shift towards 5 GHz, as the second stripline (L2-C2-R2) already shifted the higher resonance to the frequency higher than 5 GHz, due to coupling with the second stripline. It is essential to mention that the second stripline is needed to avoid unwanted resonance in the region between the wanted resonances.

#### Analysis of the performance of the finalized unit cell

**Figure 7:**
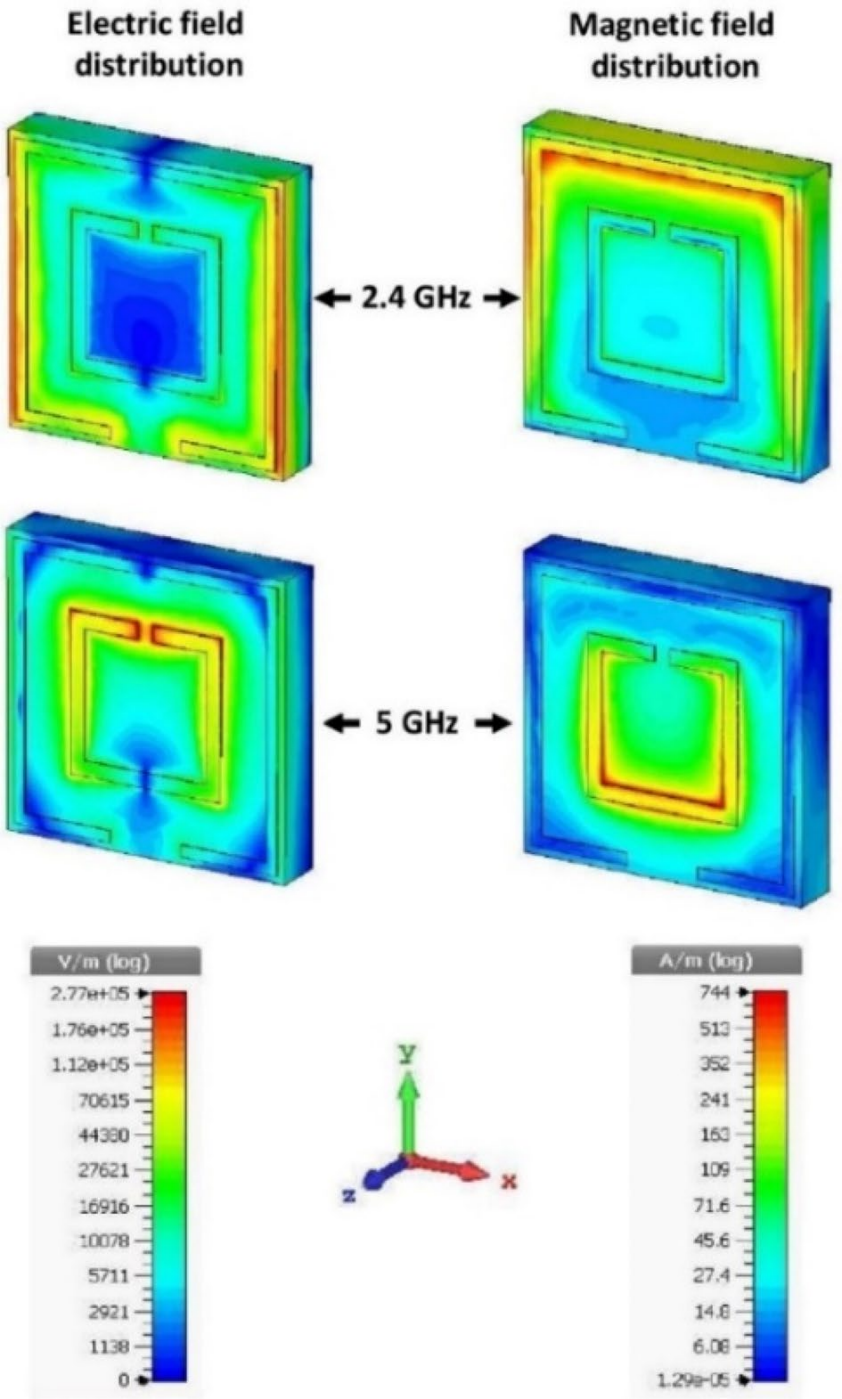
Distribution of the electric field and the magnetic field at 2.4 GHz and 5 GHz on the MM unit cell.

**Figure 8:**
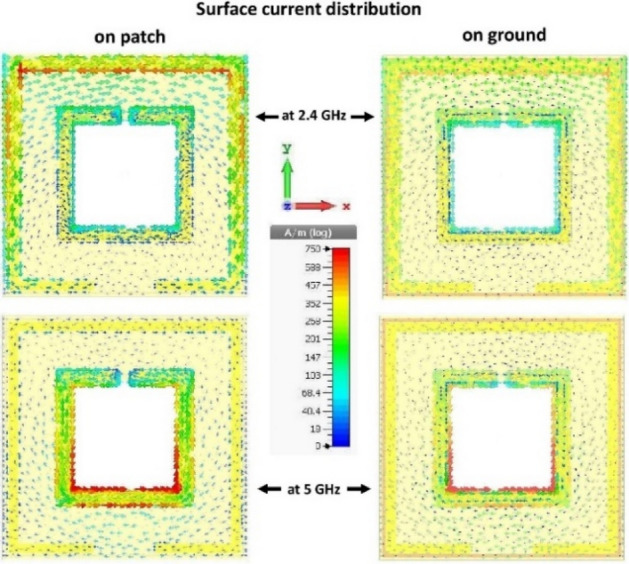
Distribution of surface current on the top (patch) and the bottom (ground) surface of the MM unit cell at 2.4 GHz and 5 GHz.

The unit cell's final design shows promising results at the desired resonance frequencies by CST simulation, as can be revealed from Figs. 7 and 8. The electric fields (and corresponding magnetic fields) show that the two SRRs act simultaneously and separately for the two desired frequencies. The circumferential SRR agitates for the lower resonance (2.4 GHz), and the central SRR agitates for the higher resonance (5 GHz), which is clear from Fig. 7. A clearer view can be seen from the surface current distribution in Fig. 8. The SRRs act as active resonators along with the ground central-cut edges, revealing the capacitive elements associated with each SRR and the necessity of the ground-cut.

**Figure 9:**
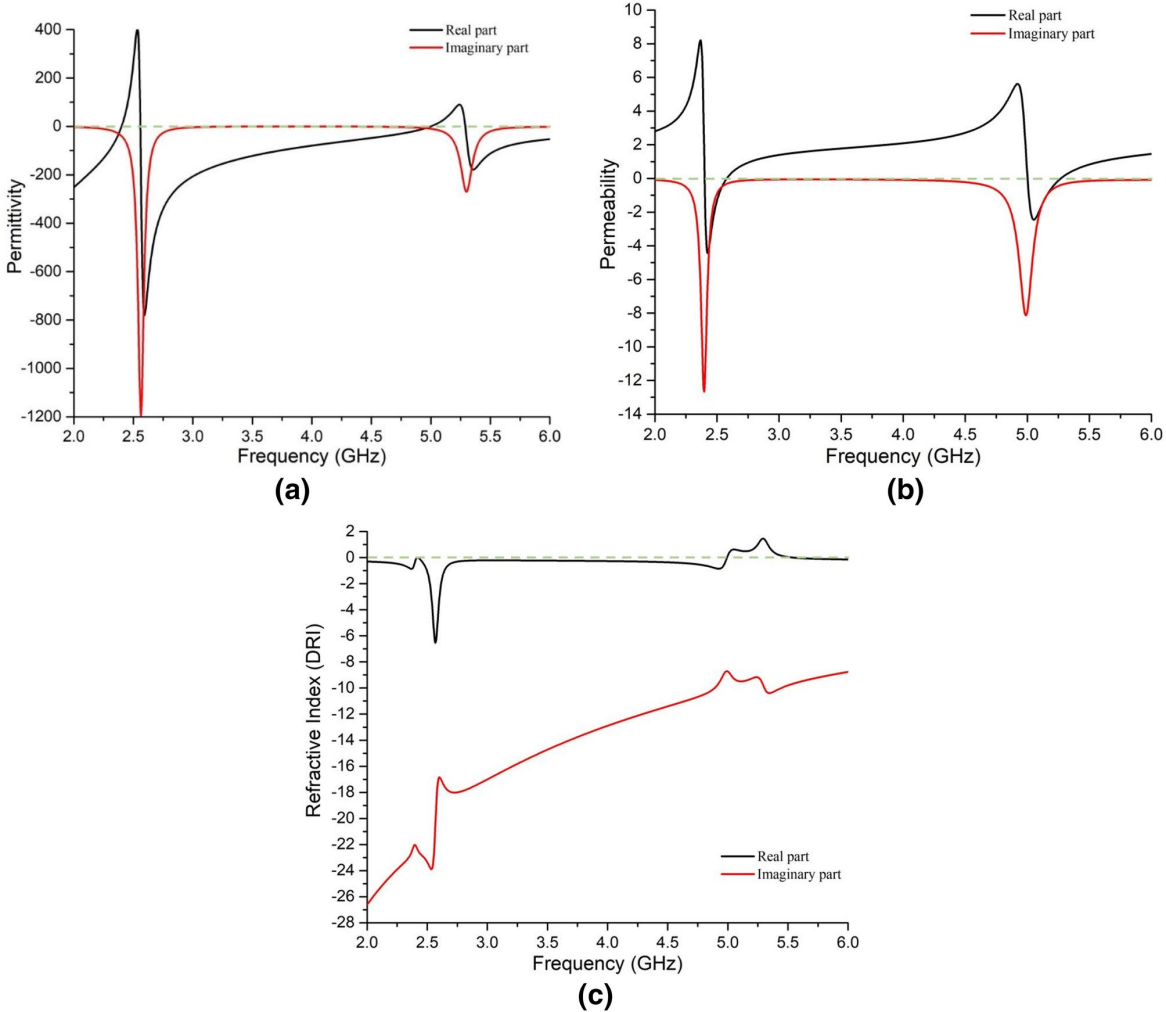
(**a**) Permittivity, (**b**) permeability, and (**c**) refractive index (DRI method) at operating frequencies.

**Figure 10:**
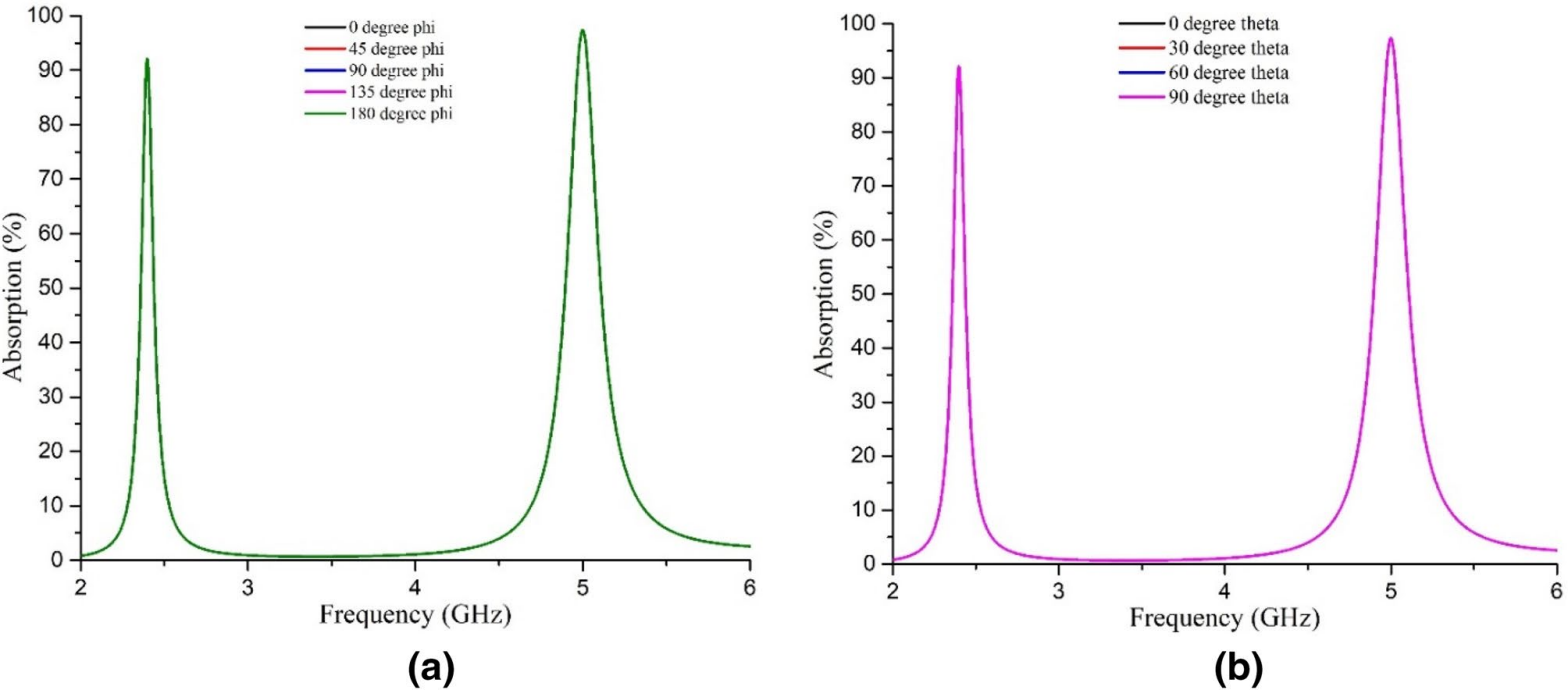
Absorption performance of the unit cell for (**a**) normal incidence and (**b**) oblique incidence.

At the resonance frequencies, the absorptivity, permittivity, permeability, and refractive index values are listed in Table 1. Permittivity, permeability, and refractive index are shown in Fig. 9. The simulation is done at all possible co-polarizing angles. The values are found similar for all incident angles of the EM wave, as can be seen from Fig. 10.

**Table 1:** Parameter values from cst simulation at the resonance frequencies for all co-polarization angles.

Parametric output	At 2.4 GHz	At 5 GHz	Co-polarization angles
Absorption	91.9%	97.35%	At all theta (0, 30, 60 & 90 degrees) polarization and phi (0, 45, 90, 135 & 180 degrees) polarization
Permittivity	− 1.12	0.099	
Permeability	0.045	0.19	
Refractive Index (DRI method)	− 0.275	0.15	
Refractive Index (NRW method)	− 0.275	− 0.15

It is observed from Table 1 that SNG value was achieved at 2.4 GHz, and near-zero values were found for 5 GHz. Thus, due to the SNG value, the unit cell can be claimed as a metamaterial (MM) unit cell.

According to complex Poynting vector^33^, when the EM waves travel from one medium to another, there will be energy flow across the boundary surface, and it can be estimated by $\overrightarrow {{P_{C} }}  = \frac{1}{2}\vec{E} \times \overrightarrow {{H^{*} }}$. According to the law of conservation of energy, the rate of energy dissipation in the first medium will be equal to the rate of energy flow in the second medium and vice-versa. In a complex medium (where imaginary parts of permittivity, permeability and refractive index are negative), the average power flow is calculated by

$$ P_{{av}}  = \frac{1}{2}~Re\left\{ {\vec{E} \times \overrightarrow {{H^{*} }} ~} \right\} = ~\frac{1}{2}~Re~\left( {EH^{*} } \right) = ~\frac{1}{2}~Re~\left( {\eta HH^{*} } \right) $$

If $H = H_{m} e^{{j\omega }} t$ and $H^{*}  = ~H_{m} e^{{ - j\omega t}}$; then

10 $$ P_{{av}}  = ~\frac{1}{2}~H_{m}^{2} ~Re~\left( \eta  \right) $$

where $H_{m}$ is the maximum value of magnetic field and ŋ $\left[ { \approx ~\sqrt {\frac{\mu }{\varepsilon }} \left( {1 + j\frac{\sigma }{{2\omega \varepsilon }}} \right)} \right]$ is the intrinsic impedance of the second medium (substrate). For dielectric medium (like FR4 substrate), the dissipation factor $(D_{f}  \equiv ~\frac{\sigma }{{\omega \varepsilon }}$) is less than 1.

According to W. C. Chew^34^, to conserve EM energy is a complex medium, the real parts of $\varepsilon$ and $\mu$ have to be negative and their imaginary parts have to positive and vice-versa. Moreover, as per Eq. (10) the average power loss in medium 1 (here the air medium above the proposed absorber) will be equal to power flow in the medium 2 (here the absorber) and it depends on the real part of ŋ. Since the real parts of $\varepsilon$, $\mu$ and refractive index at the resonance frequencies are found positive and the corresponding imaginary parts are found negative (Fig. 9), as per the above discussion the proposed absorber obeys the law of conservation of (EM) energy.

The unit cell was applied with the normal and oblique incidence of the EM waves and has shown similar absorptivity for both cases, as depicted in Fig. 10. At all polarizing angles (co-polarized), the unit cell shows more than 90% absorptivity at 2.4 GHz and 5 GHz, specified for WiFi signal, universally.

Absorption was calculated from S_11_ and S_21_ parameters by the usual formula as below:

11 $$ A = 1 - ~\left| {S_{{11}} } \right|^{2}  - \left| {S_{{21}} } \right|^{2} $$

#### Measurement of the absorber

**Figure 11:**
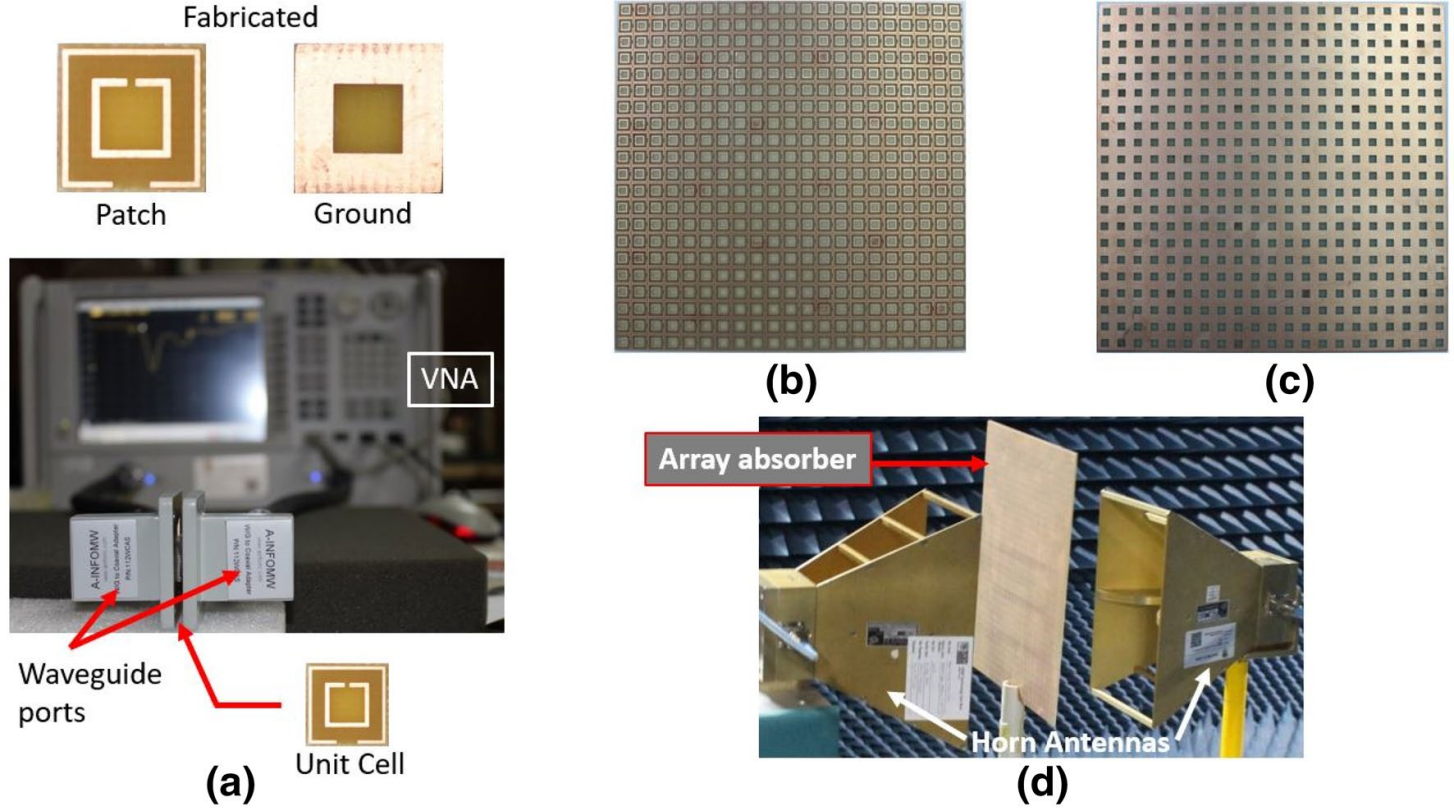
Fabricated unit cell (**a**) measurement setup of the unit cell with a vector network analyzer (VNA), (**b**) fricated array patch, (**c**) fabricated array ground, (**d**) array measurement setup in the anechoic chamber.

It is essential to fabricate the absorber and then measure it for ensuring performance according to the theoretical and simulation output. The unit cell and the array of the essential number of unit cells should undergo practical measurement purposes. A proper experimental setup with no unwanted infiltration of external EM waves is the prior criteria. Hence the array should be set up inside an anechoic chamber, ensuring external EM wave shielding. In Fig. 11, the experimental setup of measurement for both the unit cell and array with a vector network analyzer (VNA) is shown. The details of the calibration and measurement is written in the “Methods” section after conclusion.

**Figure 12:**
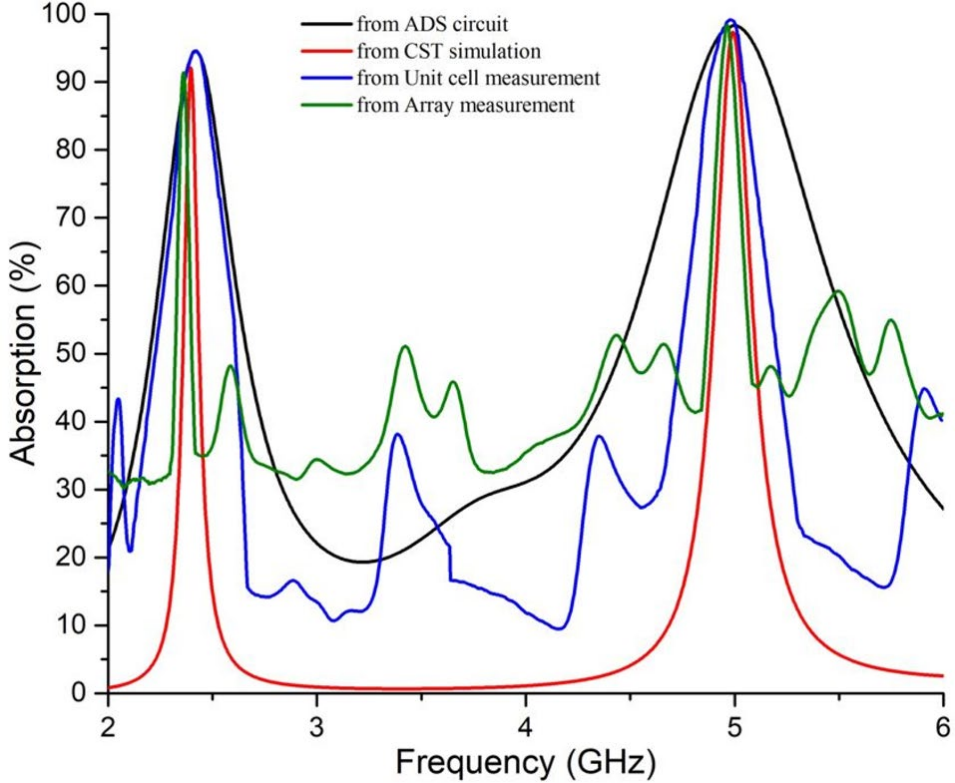
Comparison of absorption for measurement of the unit cell and the array with that of simulations.

S parameters were extracted from measured data to calculate absorption performance by the fabricated absorber as per Eq. (11). The proposed absorber has shown perfect absorption at the resonance frequencies (2.4 and 5 GHz) for both the unit cell and the array, plotted in Fig. 12.

It can be noticed from Fig. 12 that, there are some deviations in the measured data at non-resonant frequency bands. It happened because of the deduction of noises from calculated S parameters of the measurement data for both unit cell and array. During measurement, there were some aberrations in the non-resonant frequencies and insignificant aberrations at the resonance frequencies. To get optimum result, the noises in terms of such aberrations were deducted to show proper measured data, which resulted as shown in Fig. 12. Although the objective of the measurement process was to achieve absorptions at resonance frequencies and it was indeed achieved, but there are some differences in the bandwidth achieved by the simulations and measurements. It might happen due to added losses in the coaxial cable connectors with the coaxial-to-waveguide adapters. The improper impedance in the connectors may result some false absorption around the resonance frequencies that caused the broad band absorptions. Similarly, the ADS circuit has shown broad-band absorption as the ADS circuit is considered as the equivalent circuit (not exact replacement of the unit cell designed on CST).

Finally, the Fig. 12 proves that the proposed equivalent circuit model, and the subsequent procedure to design metamaterial absorber at desired frequencies, is efficient.

## Discussion

The proposed method describes a perfect way to design a co-polarization-insensitive metamaterial unit cell that can efficiently absorb WiFi signals (both 2.4 GHz and 5 GHz). The designed unit cell is based on conventional square split-ring resonators with mathematically calculated ring and spacing dimensions. These dimensions were emanated from the equations for L, C, and R by the proposed equivalent circuit theory with extensive analysis. The unit cell size is the least (0.08 λ) in terms of the wavelength corresponding to the lowest resonance frequency. It shows metamaterial characteristics at lower resonance with SNG values (Table 1). Moreover, it does not require any lumped elements^35^ or multilayer substrate^36^, present in all designs proposed by other researchers for the same purpose. The design is compact in terms of effective medium ratio (EMR = $${\raise0.7ex\hbox{${\lambda _{0} }$} \!\mathord{\left/ {\vphantom {{\lambda _{0} } L}}\right.\kern-\nulldelimiterspace} \!\lower0.7ex\hbox{$L$}}$$) with the value of 12.49 considering $$\lambda _{0}$$ for 2.4 GHz. The practical measurement results coincided with simulation to prove the design as robust. A comparison table can differentiate the proposed design's appropriateness and efficiency as per the described method, depicted in the Table 2 below.

The comparison Table 2 has proved that the proposed equivalent circuit approach is the efficient method to design metamaterial absorbers with high EMR for desired output at target resonance frequencies. Conventional split-ring-resonator (SRR)s can be used for the patch design, with precision in the design using the proposed method. Moreover, there will be no necessity for any lumped element or multilayer substrate. The proposed absorber can be utilized for various WiFi applications, like EM energy harvesting, SAR reduction for mobile devices, crowd-sensing/estimation application under WiFi coverage, WiFi signal sensing, EM coupling reduction in devices using both WiFi and LTE, etc.

**Table 2:** Comparison of the proposed wifi absorber with most relevant works.

Ref	Year	Shape of the absorber	Resonance frequencies (GHz)	Size of the unit cell (mm)	SNG /DNG values	Substrate and thickness (mm)	Maximum polarization angle insensitivity	Equivalent circuit analysis	EMR
37]	2018		2.45 & 5	(34 mm) 0.27λ	Not reported	Two-layered FR4 (3.2 mm)	Not reported	Not shown	3.67
[38]	2019		2.2–5.85	(40 mm) 0.29λ	Not reported	Multilayers ITO- PET (11 mm)	90°	Not shown	3.12
[39]	2020		2.4 & 5.1	(18 mm) 0.14λ	Not reported	Two-layered Rogers RO 3003 (1.75 mm)	90°	Simple analysis shown	6.94
Proposed	2021		2.4 & 5	(10 mm) 0.08λ	SNG	Single-layer FR4 (1.6 mm)	180°	Extensive analysis shown	12.49

## Conclusion

This article describes an efficient equivalent circuit approach to design a perfect co-polarized MM absorber for WiFi signal absorption with the least unit cell area and substrate thickness. The polarization insensitivity was proved for this design up to 180 degrees of applied EM waves' incident angle, which is unprecedented among similar absorbers to date. The proposed absorber achieved a maximum of 99% absorptions with an effective medium ratio (EMR) of 12.49, which is the maximum among all WiFi absorbers. The equivalent circuit analysis for L, C, and R was appropriate for the proposed unit cell with a partial ground, which is a new contribution to the circuit design. For simplicity of the calculation, conventional square-shaped split-ring resonators (SRR) were chosen for the patch design with precision in SRR dimensions for a proper bounding area to get desired surface resistance, R, which satisfied less than − 10 dB value of S parameters. The proposed model can be employed in mobile phones or laptops to absorb unwanted EM waves to reduce SAR to the human body. Also, the model can be utilized for EM energy harvesting applications in microwave devices like a smartwatch or wireless headphones. Finally, the unit cell engineering by equivalent circuit analysis described in this article may help researchers design future-generation efficient EM absorbers for other relevant applications.

## Methods

### Measurement

The unit cell was essentially fabricated (as shown in Fig. 3) to measure its absorbance from S parameters using an Agilent Network Analyzer N5227A with two separate waveguide ports (waveguide-to-coaxial adapters) (one for 2–3.75 GHz and the other for 3.75–6 GHz) connected to the two ports of the VNA simultaneously. For array measurement, two horn antennas setup at 180 mm distance apart from each other in an anechoic chamber were connected with coaxial cables to the VNA outside the chamber. On VNA, the number of points for the two waveguide ports were set to 430 and 573 (as per simulation) and then combined to extract absorptions to align experimental data with simulation. The VNA setup was calibrated with the e-cal method by Agilent N4694-6001 electronic calibration module for 2 to 6 GHz for the two different waveguide ports for measurement purposes outside the anechoic chamber. During calibration for both the waveguide ports and the horn antennas setup, some noises were recorded without the experimental unit cell and the array. These noises might have occurred due to the SMA connectors used with the coaxial cables to connect the waveguide ports and the horn antennas. For ease of calculation of the measured data, these noises were deducted from the readings found during unit cell measurement (with waveguide ports) and array measurement (with horn antennas). S parameters (S11 and S21) were copied to an Excel file in both real and imaginary parts. Matlab was used (with necessary codes) to calculate absorption using data from the Excel file. With features like wide-incidence, angle insensitivity for co-polarization, and SNG values, the measured values were found to be very close to simulated values, suggesting that it is a good candidate for absorption in 2.4 and 5 GHz dual-band WiFi applications.
